# Johnson–Cook Parameter Identification for Commercially Pure Titanium at Room Temperature under Quasi-Static Strain Rates

**DOI:** 10.3390/ma14143887

**Published:** 2021-07-12

**Authors:** Alice Siegel, Sébastien Laporte, Fabien Sauter-Starace

**Affiliations:** 1 Clinatec, CEA, LETI, Université Grenoble Alpes, F-38000 Grenoble, France; alice.siegel@outlook.com; 2 Institut de Biomécanique Humaine Georges Charpak, Arts et Métiers Institute of Technology, F-75013 Paris, France; sebastien.laporte@ensam.eu

**Keywords:** grade 2, commercially pure titanium, Johnson–Cook, plasticity, tensile test, microstructure

## Abstract

Background: To simulate mechanical shocks on an intracranial implant called WIMAGINE^®^, Clinatec chose a Johnson–Cook model to account for the viscoplastic behavior of grade 2 titanium in a dynamic study using Radioss^©^. Methods: Thirty tensile specimens were subjected to tensile tests at room temperature, and the influence of the strain rate (8 × 10^−3^ and 8 × 10^−2^ s^−1^) and sandblasting was analyzed. Relaxations were included in the tests to analyze viscosity phenomena. Results: A whole set of parameters was identified for the elastic and plastic parts. Strain rate influence on stress was negligible at these strain rates. As expected, the sandblasting hardened the material during the tests by decreasing the hardening parameters, while local necking occurred at an earlier strain. Conclusions: This article provides the parameters of a Johnson–Cook model to simulate the elastoplastic behavior of pure titanium (T40, grade 2) in Finite Element Model (FEM) software.

## 1. Introduction

Titanium and its alloys are widely used in the biomedical field, mainly for their corrosion resistance, biocompatibility, and high specific strength. Implants and, more recently, active implant housings are made of titanium and its alloys. Although Ti-6Al-4V is still the most used titanium alloy in this field, commercially pure titanium (CP-Ti) is sometimes preferred for long-term applications due to cytotoxicity and genotoxicity of aluminum and vanadium release [1,2,3,4]. CP-Ti is comprised of over 99% of titanium, and four grades are available, varying in carbon, iron, and oxygen amounts and acting on the corrosion resistance, formability, and ductility.

At CEA Clinatec, the intracranial wireless ECoG implant WIMAGINE^®^ was developed in a Brain–Computer Interface project [5,6]. Little mechanical load is generally applied to implants, and Hooke’s law is usually sufficient to model elastic deformations. Young’s modulus values for CP-Ti grade 2 are reported between 105 and 118 GPa in the literature [7,8,9,10]. Grain boundary sliding is reported during deformation thanks to microscopic analysis, though its impact on the overall mechanical strain is negligible [11].

To prevent excessive deformation or mechanical failure in impact situations due to falls or blows, the engineer has to model the plastic behavior of CP-Ti. Complex analysis of CP-Ti plasticity implies the modelling of twinning and texture evolution, which explains the anisotropic hardening behavior [12]. In the finite element analysis with Radioss^©^ for an intracranial implant subjected to impact, the constitutive model is simplified to an engineer’s tool, such as the isotropic Johnson–Cook (JC) model [13]. Cheng et al. [14] published the identified parameters of this model for Ti-6Al-4V. Hereafter, JC parameters were identified on samples made of commercially pure titanium (CP-Ti), and the influence of sandblasting and quasi-static strain rate variation were analyzed.

The aim of this study is therefore to propose a robust methodology to evaluate the JC mechanical parameters of commercially pure titanium and to identify the influence of sandblasting and strain rate variation on these parameters.

## 2. Materials and Methods

All following stresses and strains are true stresses and strains.

### 2.1. Experimental Setup

#### 2.1.1. Tensile Specimens

**Figure 1:**
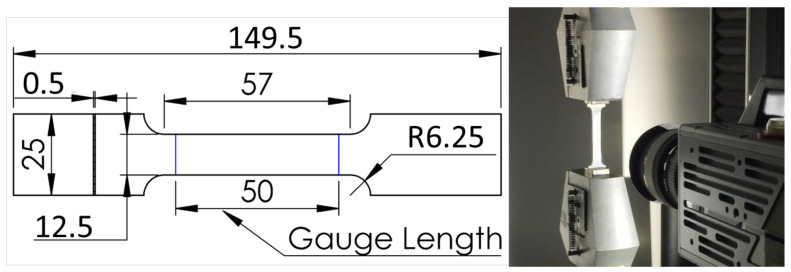
(**Left**) Specimen design following ISO 6892-1:2016; dimensions in mm. (**Right**) Set-up for tensile test.

Thirty CP-Ti tensile specimens (Micro Tolerie Dallard, Saulce sur Rhone, France) were designed following ISO 6892-1:2016 [15] (Figure 1) and cut in a 0.5 mm thick hot rolled and annealed sheet.

#### 2.1.2. Tensile Tests

Uniaxial tensile tests were performed at room temperature on a tensile machine model 5566 (INSTRON, Norwood, MA, USA) with a 10 kN load sensor. Specimens were sprayed with white and matt black paint to obtain a random grayscale pattern. A camera (Fastcam SA3, PHOTRON Inc., San Diego, CA, USA) recorded the tests at 50 fps to perform digital image correlation (DIC) [16]. All specimens were tested until failure.

The specimens were split into three groups (G1, G2, and G3). Taking into account the tensile machine limitations, two different velocities (0.5 and 5 mm/s, corresponding to 8 × 10^−3^ and 8 × 10^−2^ s^−1^ strain rates) were tested to investigate the strain rate sensitivity in the material between G1 and G2. In addition, the influence of sandblasting was analyzed between G1 and G3. Ten specimens were tested for each condition (Table 1).

Three stress relaxations were included in the plastic hardening part for G1 and G3 at 3.3, 6.4, and 9.4% of true strain (obtained from preliminary tests) to track viscoplasticity phenomena. The constant strain was held during the 30 s to observe short-term relaxation phenomena. Relaxations could not be performed on G2 specimens due to machine limits on velocity.

### 2.2. Stress–Strain Curves

**Figure 2:**
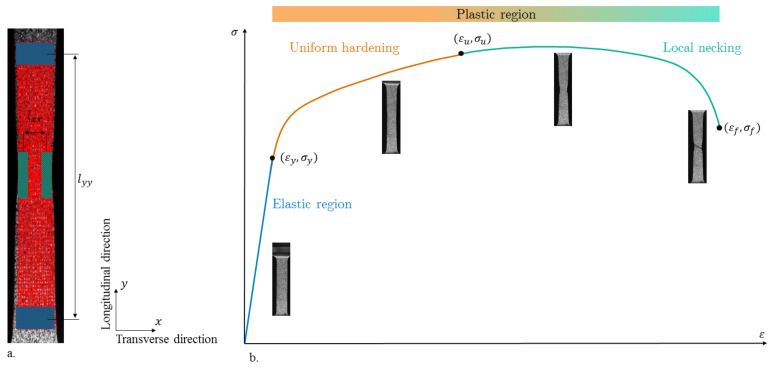
(**a**) Image analysis of the tensile test specimens. (**b**) Strain–stress curve of the grade 2 titanium.

A free Matlab^®^ code (MathWorks Inc., Natick, MA, USA) [17] enabled the tracking of the grayscale pattern motion up to failure using a 75 × 17 tracking grid (Figure 2a). Grid marker positions were extracted at each time step and used to compute logarithmic true strains ε.

Longitudinal strains (on the y-axis) were computed using the five upper and five lower ranges of grid points. Transverse strains were computed using the two right and two left columns of grid markers in the necking region.

The material behavior was assumed isotropic, and Poisson’s ratio could be considered homogeneous throughout the material. This enabled the computation of the cross-sectional area *A* using the transverse strain data obtained from DIC and the Poisson’s ratio definition. Load data *f* were recorded directly from the INSTRON machine, and true stresses σ were computed following Equation (1):

(1) $$\sigma =\frac{f}{A}$$

where *f* is the recorded load data, *A* and $A_{0}$ are the current and initial cross-sectional areas, respectively, and $l_{xx}$ and $l_{zz}$ are the current transverse lengths. $l_{xx0}$ and $l_{zz0}$ are the initial transverse lengths.

The transversal stretch $\lambda_{T}$ is defined by:

(2) $$l_{xx}=\lambda_{T}\times l_{xx0}$$

Assuming isotropic deformations in *A*, the cross section of the samples, we can express *A* as a function of $A_{0}$, $l_{xx}$, and $l_{xx0}$ as follows:

(3) $$A=l_{xx}\times l_{zz}=\lambda_{T}\times l_{xx0}\times \lambda_{T}\times l_{zz0}=\lambda_{T}^{2}\times A_{0}$$

Combining Equations (2) and (3), $A={\left(\frac{l_{xx}}{l_{xx_{0}}}\right)}^{2}\times A_{0}$

Hence, the true stress can be computed as a function of f, $A_{0}$, $l_{xx},$ and $l_{xx0}$:

(4) $$\sigma =\frac{f}{\;{\left(\frac{l_{xx}}{l_{xx_{0}}}\right)}^{2}A_{0}}$$

Grid marker positions were extracted at each time step and used to compute logarithmic true strains *ε* following Equation (5):

(5) $$\varepsilon =\ln\left(\frac{l_{yy}}{l_{yy0}}\right)$$

where $l_{yy}$ and $l_{yy0}$ are the lengths at the current and initial state, respectively.

A local strain analysis was also performed by computing the Green–Lagrange strain tensor at each grid point in the longitudinal direction (*E_yy_*), the transverse direction (*E_xx_*), and the shear direction (*E_xy_*) at each time step, as indicated in Equation (6):

(6) $$E=\left[\begin{array}{cc} E_{xx} & E_{xy}\\ E_{xy} & E_{yy} \end{array}\right]=\frac{1}{2}\left(F^{T}F-I\right)$$

where ***F*** is the Green–Lagrange strain tensor and ***I*** is the identity tensor.

The Von–Mises effective strain $E_{VM}$ was also computed according to Equation (7):

(7) $$E_{VM}=\frac{2}{3}\;\sqrt{E_{xx}{}^{2}+E_{yy}{}^{2}-E_{xx}E_{yy}+3E_{xy}{}^{2}}$$

### 2.3. Mechanical Parameters Identification

#### 2.3.1. Data Processing Methodology

Concerning further model fitting procedures, for each specimen group, the data were processed on the whole dataset at once. All parameters were identified using a robust nonlinear least squares optimization algorithm developed in Matlab^®^. The evaluation errors on each identified parameter due to experimental noise were identified using a bootstrap method [18].

#### 2.3.2. Ultimate and Failure Stresses and Strains

The ultimate stress and corresponding strain ($\sigma_{u}$ and $\varepsilon_{u}$) were computed as the stress and strain at maximal load. Failure stresses and strains ($\sigma_{f}$ and $\varepsilon_{f}$) were not extracted for each specimen (Figure 2b) because post-necking the assumption of uniform deformation was no longer valid.

#### 2.3.3. Stress–Strain Model Fitting

Two separate models were used for the elastic and plastic hardening parts to fit the experimental curves. The elastic part was described by a 1D Hooke’s law, which is given in Equation (8):

(8) $$\sigma =E\times \varepsilon_{e}$$

where *E* is Young’s modulus to be identified and σ and $\varepsilon_{e}$ are the true stress and elastic true strain, respectively.

The JC constitutive model was chosen to describe the plastic hardening part. Relaxation data were removed from G1 and G3 raw data for this analysis. Necking is not permitted by the JC law; consequently, data after ($\varepsilon_{u}$, $\sigma_{u}$) were not considered for identification. The JC model follows Equation (9) [13]:

(9) $$\sigma =\left(\sigma_{y}+B\varepsilon_{p}{}^{n}\right)\left(1+C\ln\left(\frac{\dot{\varepsilon }}{{\dot{\varepsilon }}_{0}}\right)\right)\left(1-{\left(\frac{T-T_{room}}{T_{melt}-T_{room}}\right)}^{m}\right)$$

where *σ_y_*, *B*, *C*, and *n* are the material constants to be identified, *σ* is the true stress, *ε_p_* is the plastic true strain ($\varepsilon_{p}=\varepsilon -\frac{\sigma }{E}$), $T_{room}$ is the room temperature, $T_{melt}$ is the melting temperature, and $\dot{\varepsilon }$ and $\dot{\varepsilon_{0}}$ are the actual and reference strain rates, respectively.

It can be noticed that the two models meet at the yield stress *σ_y_*. The yield strain *ε_y_* was also computed.

The JC model was identified on each test group separately to account for behavior differences in hardening. As the strain rate is constant and the experiments were performed at room temperature, the JC model leads to Equation (10), also known as the Ludwik’s model [19]:

(10) $$\sigma =\sigma_{y}+B\varepsilon_{p}{}^{n}$$

Parameter *C* was identified using G2 data and previously identified G1 elastic and hardening parameters. The reference strain rate was ${\dot{\varepsilon }}_{G1}$.

#### 2.3.4. Relaxation Model Fitting

Stress relaxation data were analyzed using a generalized Maxwell model for the elastic modulus Equation (11) [20]:

(11) $$E\left(t\right)=E_{\infty }+\overset{N}{\underset{k=1}{\sum }}E_{k}e^{-\frac{t}{\tau_{k}}}$$

where $E_{k}$ and $\tau_{k}$ are the *k*^−th^ modulus and relaxation time, respectively, to be identified.

Two elements were chosen for the Maxwell model to account for short-term and long-term relaxation phenomena. The true stress during relaxation is given by Equation (12):

(12) $$\sigma \left(t\right)=E\left(t\right)\times \varepsilon_{R}$$

where $\varepsilon_{R}$ is the relaxation strain and *t* is the time.

The parameters computed for each specimen group are summarized in Table 2.

### 2.4. Microstructural Analysis

X-ray diffraction (XRD, Empyrean PANalytical instrument, Almelo, The Netherlands) and X-ray profile line analysis (XPLA) (X’pert HighScore Plus software, version 3.0, PANalytical, Almelo, The Netherlands) were performed at the Nano-Characterization Platform (PFNC) (MINATEC, Grenoble, France) to correlate the macro-results with micro-structural phenomena. The correction of the instrumental profile was performed first.

The used X-ray source was a Cu Kα radiation, with a wavelength λ = 1.54 Å. For these measurements, two identical post-failure G1 specimens were analyzed (referred to as TS1 and TS2). Microstrains were extracted from X-ray diffraction at distance to the rupture point (from 0 to 55 mm).

Phase analysis was performed to confirm that the sheet was only comprised of alpha-titanium (HCP—hexagonal close-packed). A texture analysis looked at the peak intensities to investigate possible privileged crystal direction.

Residual stress measurements were eventually performed using the sin2ψ method [21], based on the variation of stress concerning the tilting angle ψ.

## 3. Results

**Figure 3:**
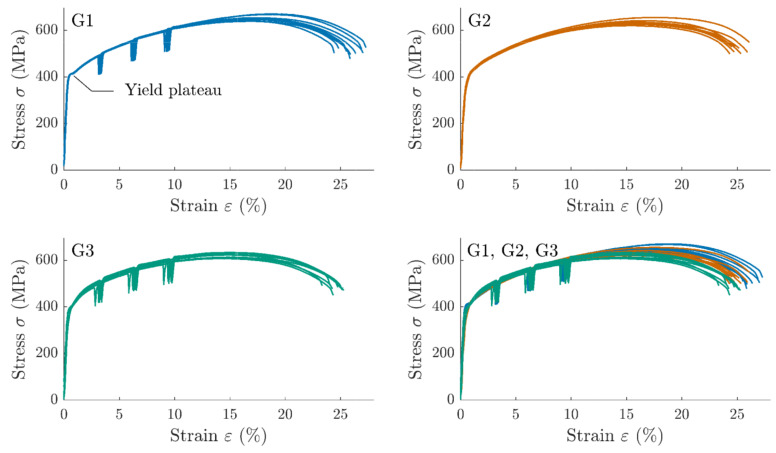
Strain–stress curves of tensile tests with relaxation for the G1, G2, and G3 groups.

Due to recording issues during the tests, one specimen was excluded from G2 and two from G3. Each of the three groups showed low dispersion before necking and low variability between groups (Figure 3). It can be noticed that G1 specimens had a yield plateau, unlike G2 and G3, for which the elastoplastic transition was more continuous.

### 3.1. Mechanical Parameters Identification

#### Ultimate and Failure Stresses and Strains

Experimental ultimate stresses and strains are summarized in Table 3. Ultimate stresses were highest for G1 and lowest for G3. Corresponding mean strains were found to be similar between G1 and G2 data, but a substantial difference can be observed between G1 and G3.

### 3.2. Stress–Strain Model Fitting

**Figure 4:**
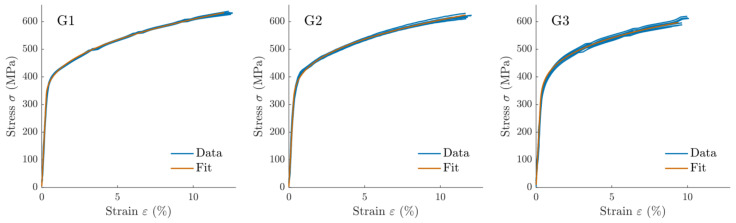
Comparison of experimental data and model fit for strain–stress curves of tensile tests limited to elastic and uniform hardening regions for G1, G2, G3 groups.

The algorithm successfully captured the elastic and plastic phenomena (Figure 4). For G1 specimens, the fit did not recreate the yield plateau during the transition from elastic to plastic behavior, which JC’s constitutive law cannot model.

**Figure 5:**
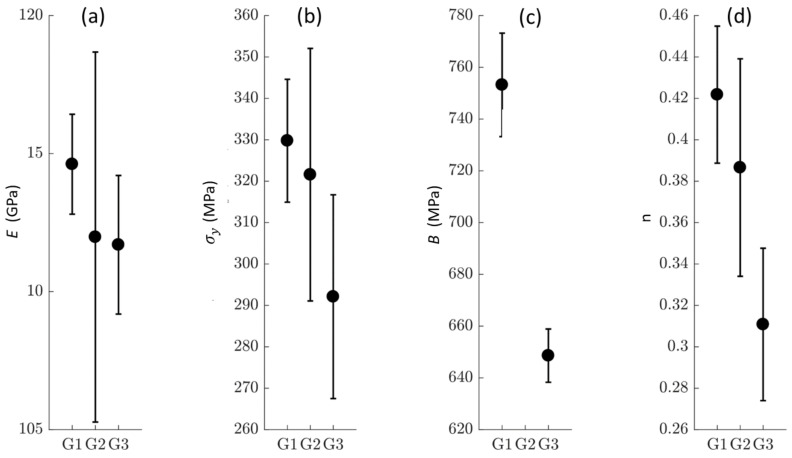
Identified parameters for the three groups. (**a**) *E* the Young’s modulus, (**b**) $\sigma_{y}$ the yield stress, (**c**) *B* the hardening parameter, (**d**) n the hardening coefficient.

The mechanical parameters obtained through the robust algorithm are presented in Table 4, and the mean values comparing the three groups are shown in Figure 5. The identified Young’s moduli, yield stresses, and yield strains were similar between groups. Standard deviations were low for G1 and highest for G2. The value of parameter *C* was in the range of $10^{-13}$.

Concerning the hardening parameter *B*, it was highest for G1 data, with a mean value of 753 MPa. Both G2 and G3 results showed a significant difference with G1, with mean values of 707 and 649 MPa, respectively. It can be noted that the G3 data had a more important difference. Eventually, no difference could be noticed for n between G1 and G2, while there was a substantial difference between them for G1 and G3, with mean values of 0.42 and 0.31, respectively.

The rate parameter *C* (~10^−13^) indicated no influence of the strain rates on such a small range.

#### 3.2.1. Local Strains

**Figure 6:**
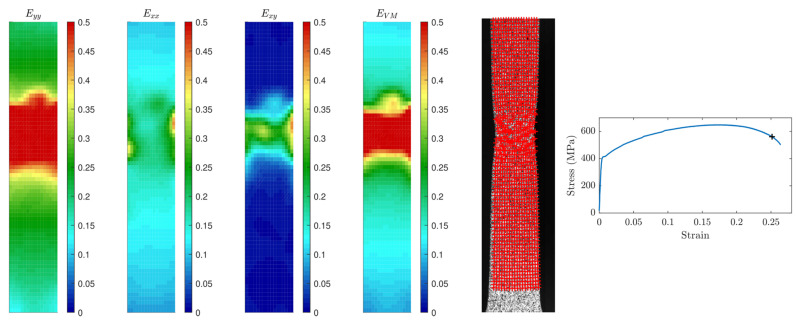
Strain representation ((**a**) $E_{yy}$, (**b**) $E_{xx}$, (**c**) $E_{xy}$, (**d**) $E_{VM}$) on a specimen, (**e**) grid representation of sample after plastic deformation. (**f**) Strain–stress curve showing elastoplastic behavior.

Local strains were analyzed step by step for each tensile specimen, and a screenshot of the analysis is given in Figure 6. After little motion during the elastic part, longitudinal $E_{yy}$ strains started to grow. The localized necking is pictured by $E_{xx}$ and $E_{xy}$ increases and localization of the four strain maps. Relaxation data were also investigated for new organizations of the lattice, which was not observed in the dislocation analysis and seemed to confirm that viscoplastic phenomenon is not based on dislocations glide or increase.

#### 3.2.2. Stress–Time Model Fitting

**Figure 7:**
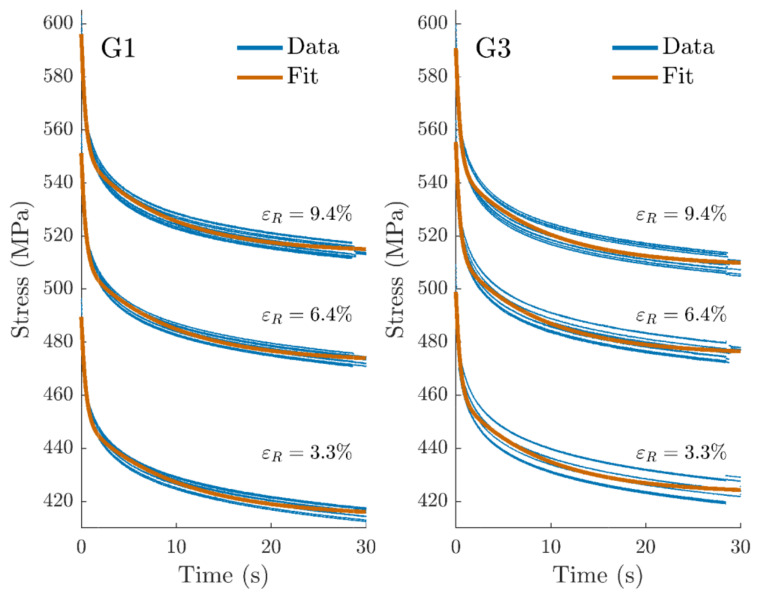
Time–stress curves related to relaxation tests for groups 1 and 3.

**Figure 8:**
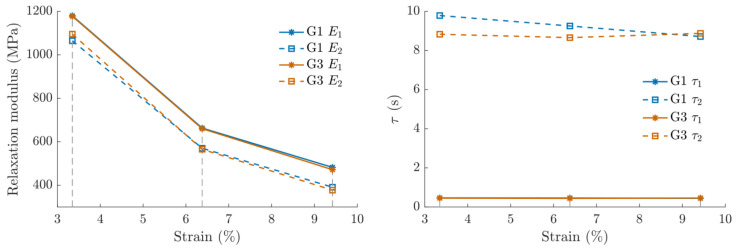
For groups 1 and 3 (**Left**) Strain–relaxation modulus. (**Right**): Strain–relaxation time.

The relaxation moduli and times were identified for all three relaxations on G1 and G3 data. R^2^ values of 0.95 and 0.78 were found for G1 and G3 fits, respectively; the difference in fit quality was mainly due to a higher data variability in G3. The experimental data and fitted curves are shown in Figure 7, and identified parameters can be found in Table 5. No difference could be found for any of the parameters between groups G1 and G3. Relaxation moduli decreased over the relaxation strains, whereas relaxation times $\tau_{1}$ and $\tau_{2}$ stayed constant one by one over the three relaxations (see Figure 8).

### 3.3. Microstructural Analysis

The phase analysis of the samples confirmed that all of them exhibited only the titanium HCP alpha phase. The texture analysis revealed no preferred direction.

**Figure 9:**
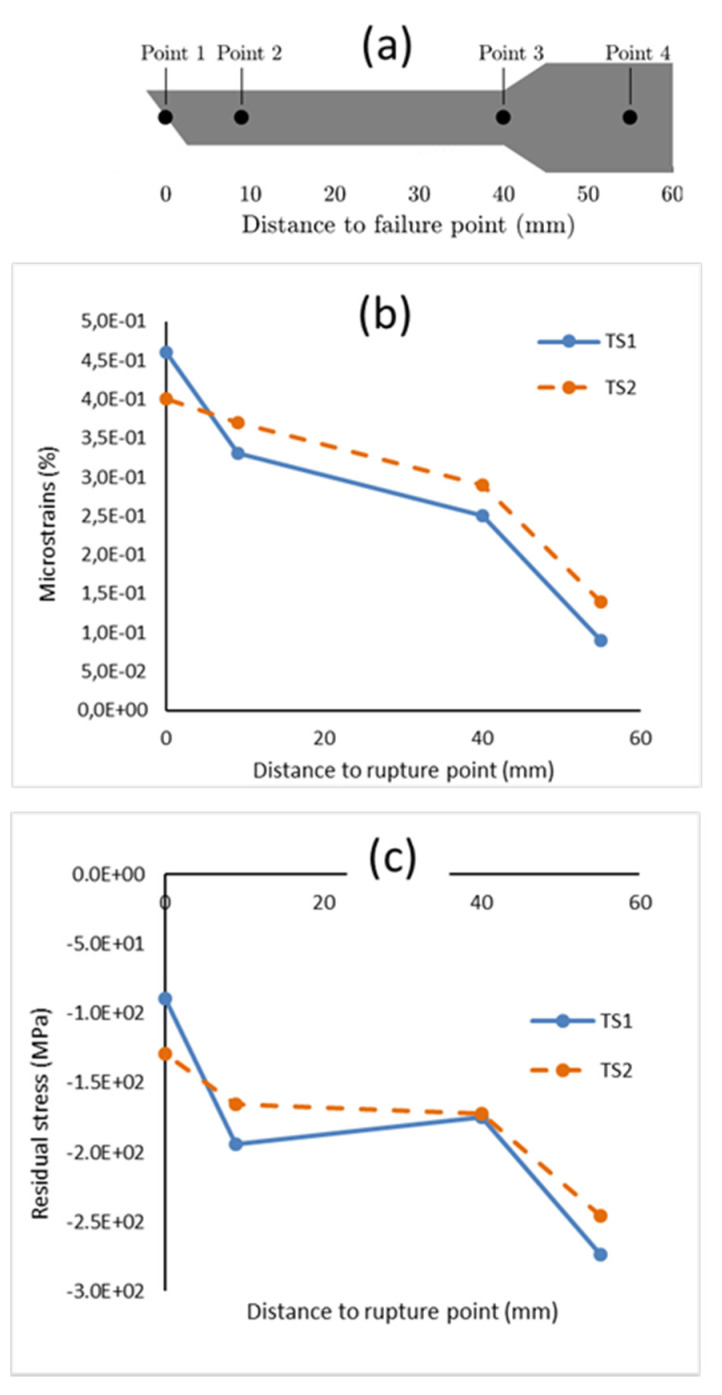
(**a**) Location of the characterization area on the tensile specimen after failure; (**b**) estimations of microstrains; (**c**) residual stress as a function of the distance to the failure line. TS1 is in blue and TS2 is in orange.

Residual stresses in the tensile direction in both TS1 and TS2 specimens are depicted in Figure 9c. The clamped regions depicted the highest compressive stresses, while the failure points showed the lowest residual stresses and the highest microstrains.

## 4. Discussion

**Figure 10:**
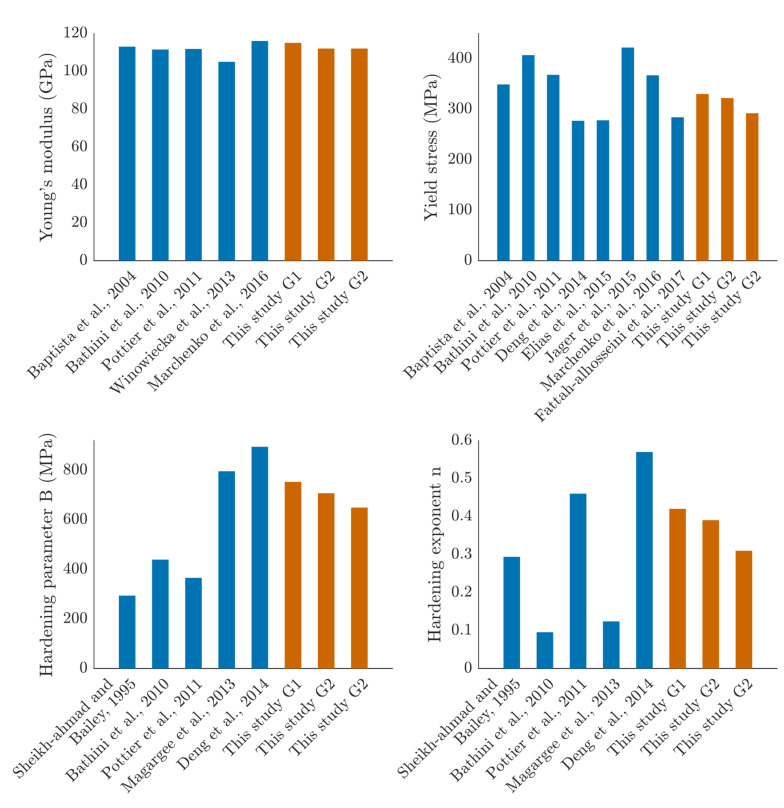
Comparison to the literature of the identified parameters (**a**) *E* the Young’s modulus, (**b**) $\sigma_{y}$ the yield stress, (**c**) *B* the hardening parameter, (**d**) n the hardening coefficient.

As previous authors have already identified the JC model parameters for commercially pure titanium (CP-Ti) [10] or Ta6V [14], this paper does not claim originality. The present study aimed to provide a complete set of mechanical parameters for the elastic and plastic behavior of the CP-Ti and to evaluate the influence of quasi-static strain rates and sandblasting. In Figure 10, the model parameters identified in our three groups are compared to the literature.

Young’s modulus and yield stress are in the range of the previously identified values [7,8,10,22,23,24,25]. In this work, 292 < *B* < 330 MPa, meaning *B* is lower than previously published values. The choice of identification method may justify this, since parameters *E, A, B*, and n are identified through a robust fitting algorithm from the joint Hooke and JC models at once. This limits the fitting errors of the plastic fit depending on the elastic one and improves the overall model fit compared to the 0.2% offset method, which is the convention usually applied, such as by Johnson and Cook when first describing their method in 1983.

Literature values [7,26,27,28] on *B* and n hardening parameters are very variable (Figure 10c,d, respectively). However, such variability for the strain hardening parameters influences the outcome of plastic strains and stresses in modeling plastic deformations highly.

The effect of sandblasting was found on *B* and n parameters by decreasing their values concerning G1 parameters. A decrease in n stands for a strengthened strain hardening, while a decrease in *B* leads to the maximal load being reached earlier on. This was the case here, as the ultimate stresses were reached at 12.4% strain for G1 and 9.9% for G3. Therefore, the surface treatment of sandblasting strengthens the material in the strain hardening part, though limiting its deformation capacity.

In this work, the strain rate range is too small to identify correctly parameter *C*. The use of Split Hopkinson Pressure Bar tests allows the reaching of strain rates between 10^−3^ s^−1^ and 6000 s^−1^ for which two studies found *C* = 0.06 at high strain rates [26,27].

Anisotropy of titanium related to rolling was not considered, though it is reported in the literature to have a significant influence in the parameter values [7,8]. Yet, the present XRD analyses reveal no texture and stress annealing, meaning the material is without preferred direction.

The XPLA confirmed an increase of plastic microstrains in the failure area by the local strain analyses. HCP metals, in general, depict more complex plasticity mechanisms than FCC (face-centered cubic) or BCC (body-centered cubic) crystals. Salem et al. [29] also indicated that high-purity titanium deforms plastically at room temperature by twinning and slip through three distinct mechanisms: dislocations slip, Hall–Petch grain size strengthening, and texture softening.

The extraction of the dislocation densities was not consistent using the Williamson Hall’s model [30] or the Gay’s model [31]. Hence, the evolution of dislocation densities should be confirmed by complementary analysis like Transmission Electron Microscopy (TEM).

The relaxation moduli decreased for increasing relaxation strains, while relaxation times stayed constant. No reference to this phenomenon has been found in the literature, and future investigations could give insight into the mechanisms involved. It indicates that during the strain hardening phenomenon, the relaxation capacity of the material decreases.

## 5. Conclusions

Thanks to a new experimental campaign on commercially pure titanium grade 2 for quasi-static strain rates and room temperature with a dedicated and modern identification strategy, we have identified a set of strain hardening parameters for a Johnson–Cook model for grade 2 titanium. This model can be implemented into a finite element software, such as Radioss^®^. Relaxation data are given for modeling short and medium-term viscosity phenomena.

## Figures and Tables

**Table 1:** Test conditions for each specimen group.

Specimen Group	Strain Rate (s^−1^)	Sandblasting	Relaxations
G1	8 × 10^−3^	-	X
G2	8 × 10^−2^	-	-
G3	8 × 10^−3^	X	X

**Table 2:** Computed parameters for each specimen group.

Specimen Group	$\left(\varepsilon_{y},\sigma_{y}\right)$	$\left(\varepsilon_{u},\sigma_{u}\right)$	*E*, *B*, *n*	*C*	$(E_{k}$ $,\;\tau_{k})$
G1	X	X	X	-	X
G2	X	X	X	X	-
G3	X	X	X	-	X

**Table 3:** Experimental ultimate stresses and strains (mean and standard deviation (SD)).

	$\sigma_{u}\;(\mathrm{MPa})$			$\varepsilon_{u}\;(\%)$		
Group	G1	G2	G3	G1	G2	G3
Mean	631	619	604	12.4	11.9	9.9
SD	3	6	9	0.2	0.2	0.2

**Table 4:** Identified elastic and plastic hardening parameters.

**Mechanical** **Parameters**	**E (GPa)**			$\sigma_{y}$ **(MPa)**		
Group	G1	G2	G3	G1	G2	G3
Mean	115	112	112	330	322	292
SD	1	3	1	7	15	12
**Mechanical** **Parameters**				$\varepsilon_{y}$ **(%)**		
Group	-			G1	G2	G3
Mean	-			0.29	0.29	0.26
SD	-			0.7 × 10^−2^	2.0 × 10^−2^	1.2 × 10^−2^
**Mechanical** **Parameters**	***B* (MPa)**			**n**		
Group	G1	G2	G3	G1	G2	G3
Mean	753	707	649	0.42	0.39	0.31
SD	10	9	5	0.02	0.02	0.02

**Table 5:** Identified Maxwell parameters for relaxation phenomena.

	$\varepsilon_{R}=3.3\%$							
	$E_{1}$ **(MPa)**		$\tau_{1}$ **(s)**		$E_{2}$ **(MPa)**		$\tau_{2}$ **(s)**	
	G1	G3	G1	G3	G1	G3	G1	G3
Mean	1180	1179	0.47	0.49	1064	1094	9.8	8.8
SD	23	52	0.02	0.55	5	21	0.2	0.7
	$\varepsilon_{R}=6.4\%$							
	$E_{1}$ **(MPa)**		$\tau_{1}$ **(s)**		$E_{2}$ **(MPa)**		$\tau_{2}$ **(s)**	
	G1	G3	G1	G3	G1	G3	G1	G3
Mean	662	660	0.46	0.44	570	566	9.2	8.7
SD	14	24	0.02	0.5	4	9	0.2	0.4

## Data Availability

The data that support the findings of this study are available from the corresponding author, upon reasonable request.
